# Risk areas for the occurrence of leprosy in border countries of South America - Brazil and Argentina

**DOI:** 10.1371/journal.pone.0276977

**Published:** 2022-11-23

**Authors:** Reinaldo Antonio Silva-Sobrinho, Keurilene Sutil Oliveira, Enrique Jorge Deschutter, Marcos Augusto Moraes Arcoverde, Ismael Hoare, Ricardo Izurieta, Adriana Zilly, Maria Luzia Topanotti, Ana Maria de Almeida, Mara Cristina Ripoli Meira, Larissa Djanilda Parra da Luz, Laiz Mangini Cicchelero, Fatima Zimermann

**Affiliations:** 1 Program in Public Health in Border Region—Master’s Degree, Western Paraná State University, Foz do Iguaçu, Paraná, Brazil; 2 Master’s Degree in Public Health and Communicable Diseases, College of Pharmacy and Biochemistry, National University of Misiones, Posadas, Misiones, Argentina; 3 Public Health Doctoral Program, College of Public Health, University of South Florida, Tampa, Florida, United States of America; 4 Department of Maternal-Infant Nursing and Public Health, University of São Paulo, Ribeirão Preto, São Paulo, Brazil; 5 Department of Epidemiological Surveillance, Municipal Health Office, Foz do Iguaçu, Paraná, Brazil; 6 Department of Epidemiological Surveillance, Hospital Samic of Puerto Iguazú, Puerto Iguazú, Misiones, Argentina; Oswaldo Cruz Foundation, BRAZIL

## Abstract

**Objective:**

The aim was to analyze the spatial association and relative risk (RR) of leprosy cases diagnosed in southern Brazil and in the Argentinean province of Misiones during 2010 to 2016.

**Methods:**

This ecological-type epidemiological study analyzed data from the Health Ministries of both countries. The analysis included frequency measures, spatial autocorrelation, RR cluster analysis and map construction.

**Results:**

A hyperendemic occurrence was identified in all study regions, in the state of Paraná 71.2% of the municipalities were hyperendemic and in Misiones, Argentina 41.2%. The GI* statistical analysis showed clusters of high incidence rates in the state of Paraná and low-risk clusters in much of the state of Rio Grande do Sul, both in Brazil. The analysis indicated an area with RR equal to 3.87 - (*p* < .0001) when considering the entire territory and an RR of 2.80 - (*p* < .0001) excluding the state of Paraná, with the number of departments of Misiones, Argentina included in the risk clusters increasing significantly.

**Conclusions:**

The findings indicate a high probability of similar illness in adjacent areas, according to their relative position in space, as the occurrence of the disease is influenced by neighboring clusters.

## Introduction

Over the previous three decades, strategies have been sought to reduce leprosy cases and control this public health problem on a global scale [1]. The theme is extremely relevant and it was highlighted on the international agenda, as one of the commitments outlined in the Sustainable Development Goal (SDG) 3 of the United Nations [2]. After the 1991 adoption of multidrug therapy as a specific treatment, the World Health Organization (WHO) proposed the elimination of leprosy as a public health problem. WHO defined elimination when the prevalence of leprosy was less than 1 case per 10,000 inhabitants, and set 2000 as the global target year [3]. However, the goal was not achieved by the proposed deadline in the countries that had the most cases of the disease. In Brazil, the prevalence rate reached 4.71 cases per 10,000 inhabitants in 2000, far from the agreed indicator. From 2001 to 2018 the prevalence decreased from 3.99 to 1.48 cases per 10,000 inhabitants, with important advances in reducing the prevalence and improving the percentage of cured cases. Despite all efforts and campaigns, the expected result in relation to prevalence had not been achieved at the end of 2020, a date agreed upon after 2000 [3,4]. Therefore, as a signatory of the commitments assumed with the SDGs [2], Brazil made an effort to eliminate leprosy as a public health problem by the end of 2022 [4].

Between 2014 and 2018, Brazil diagnosed 140,578 new cases of leprosy, and during this period the country had a mean detection rate of 13.64 new cases for every 100,000 inhabitants [4]. In Argentina, leprosy is no longer considered a public health problem, however, it is one of the Latin American countries with the presence of the disease and with a heterogeneous distribution in the national territory [5], with a prevalence of 0.18 cases per 10,000 inhabitants and detection rate of 0.84 cases per 100,000 inhabitants for the year 2018 [6,7]. However, the province of Misiones, Argentina showed a prevalence rate of 0.56 cases per 10 thousand inhabitants and a detection rate of 3.07 cases per 100,000 inhabitants between 2010 and 2016. The department of Posadas, capital of Misiones province, presented a standardized detection rate of 88.75 cases/100,000 inhabitants, in the same period, classified as hyperendemic by the Ministry of Health of the Argentine [6,7].

The cities of Foz do Iguaçu-Brazil, Porto Iguaçu-Argentina and Cidade do Leste-Paraguay are the major cities of the triple frontier of these three nations. This main triple border of South America has a population of 1,100,000 inhabitants comprising of more than 72 ethnic groups. Not to mention the indigenous groups, who are some of the most vulnerable in terms of access to healthcare. The region’s economy is based on tourism, consumption of goods and services, trade in electronic products, and transit routes for products sold by the Mercosur countries [8]. This region is considered the most important border point between the province of Misiones, Argentina and Paraguay (Posadas-Encarnación); and is a major contributor to socioeconomic relations and for the health-disease processes for populations in both countries.

Among the geospatial areas for the study of health situations, border regions stand out for a number of reasons including the difficulty of controlling communicable diseases such as leprosy in these locations, impact of international territorial limits, and due to the constant population flow across borders [9,10]. The Brazilian border areas, as well as others in Latin America, must be studied from a different perspective compared to other regions, as they have specificities and particularities that often go unnoticed [10,11]. In these regions, the flows of people, goods and social relations are more complex and economic and cultural diversity generates a potential richness that is rarely considered. For example: the co-existence of many cultural groups in a relatively small area, daily life for residents of this region and its relationship with the health-disease process; the experiences of residents related to the organization of different health systems in each country; the political-economic factors and their impact on the health and social scenario. These are factors that justify the development of studies to recognize the border reality, including neglected diseases.

Considering the need for strategic information on the health status related to leprosy between international spaces that have achieved the goal of elimination and areas in transition, the use of spatial epidemiology and geoprocessing of data were chosen to allow for the visualization of interregional differences in indicators and description of the spatial distribution of the phenomena linked to leprosy. The objective of this study was to analyze the spatial association and relative risk (RR) of leprosy cases diagnosed in the period 2010 to 2016, in the South American territories of Brazil and Argentina.

## Materials and methods

**Fig 1:**
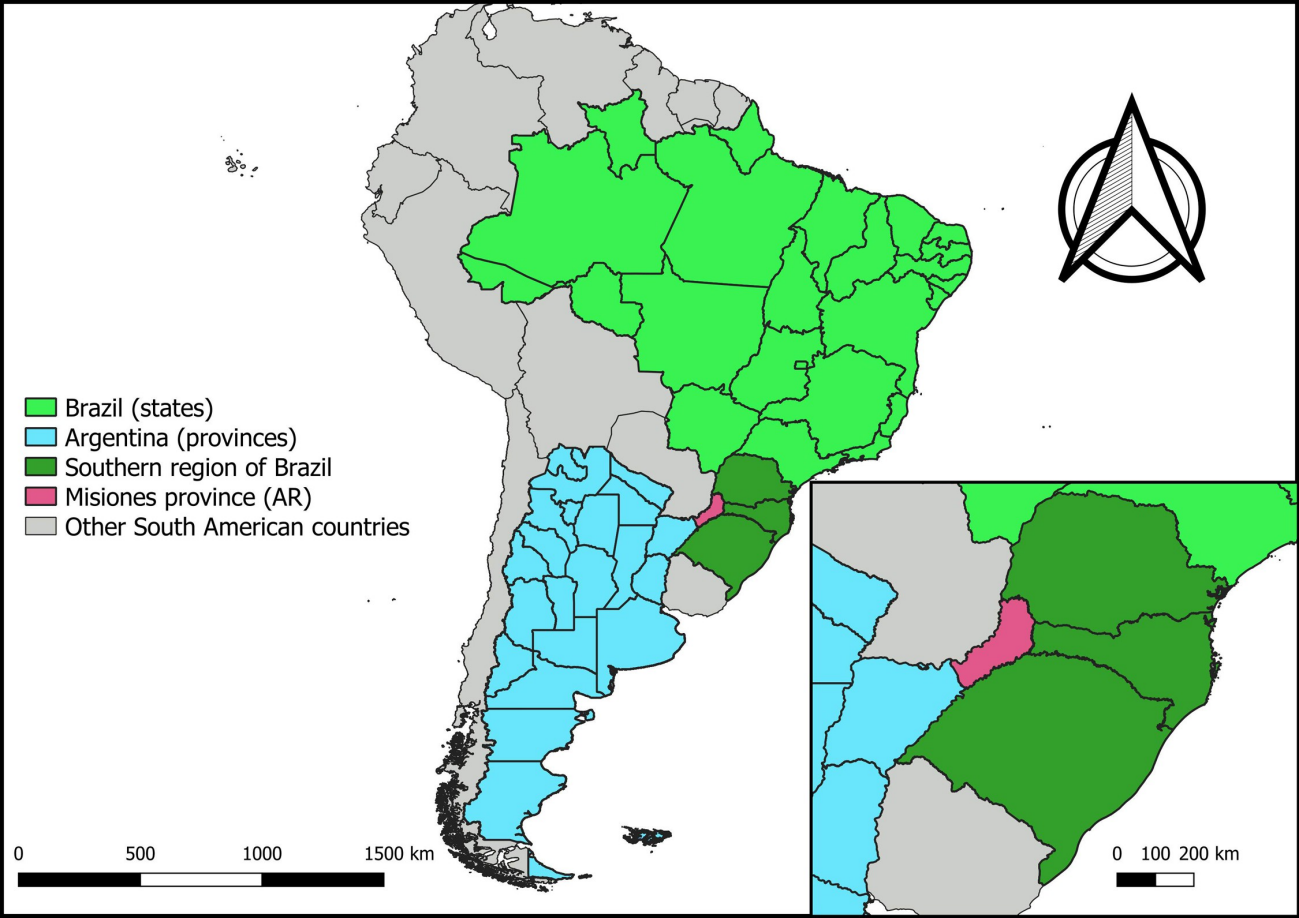
Geographic location of the southern region of Brazil and Misiones province, Argentina, 2019. Source: Oliveira, et al., 2019 [7].

This ecological-type epidemiological study involved the states of the southern region of Brazil and the province of Misiones in Argentina (Fig 1).

The southern region of Brazil consists of three states: Paraná, which had an estimated population of 11,597,484 inhabitants for the year 2021, residing in 399 municipalities, and has an area of approximately 199,307,939 km². Santa Catarina has 295 municipalities and a territory of 112,872 km², with an estimated population of 7,338,473 million in 2021. Rio Grande do Sul is located in the extreme south and is divided into 497 municipalities, with a total area of 281,730,223 km² and a population estimated for 2021 of 11,466,630 inhabitants [12]. The Brazilian health sector is made up of a mixed health system, with a universal public system, financed by public taxes (called the *Sistema Único de Saúde*) and a supplementary private system [13].

Argentina consists of 23 provinces and the capital is called the Autonomous City of Buenos Aires. Each entity has its own constitution; however, they coexist in a federal system. The provinces are divided into departments, except for the province of Buenos Aires, which is divided into parties [14]. Argentina’s health sector is structured around three main subsectors: the public subsector with public funding and provision, the mandatory social insurance subsector, and finally, the private subsector [15].

Misiones province is located in the Northeast region of the country. Almost all of its territorial limits are constituted by rivers and more than 80% are international borders, to the North and East with Brazil (Paraná, Santa Catarina and Rio Grande do Sul) and Paraguay, and to the south with the province of Corrientes- Argentina. It is politically organized into 17 departments, divided into 75 municipalities, with a total area of approximately 29,801 km², with a population of 1,175 million in 2014 [16].

The study population consisted of 10,319 new cases of leprosy (International Statistical Classification of Diseases and Related Health Problems—ICD 10 from A30.0 to A30.9) diagnosed in the three states of southern Brazil and in the province of Misiones, Argentina, with reporting years from 2010 to 2016 eligible. The data search was carried out in the first half of 2018. For the Southern Region of Brazil, the data were obtained from the Electronic System of the Citizen Information Service (e-SIC) provided by the Ministry of Health, Brazil. For the Misiones province, data were obtained in 2018 and made available by the Health Ministry of Misiones, Argentina.

In the descriptive analysis, data were presented as absolute and relative frequency. For the spatial association autocorrelation, the GI* test was employed as the statistical tool, using the GeoDa, version 1.6.7 software [17]. Then, the calculation of the relative risk (RR) of the municipalities/departments was performed, looking for agglomeration of these RRs, that is, RR clusters. This step that was performed with the SatScan, version 9.4 software, using an isotonic approach that differentiates RR levels within the same cluster. From this analysis, it was possible to recognize the highest RR as the center of each identified area, demonstrating that even within the cluster, the RR may not be the same for all municipalities. The discrete Poisson distribution was used for this calculation, seeking only high-risk clusters, without overlapping geographic clusters, circular clusters, and with 999 simulations [18,19]. Regarding the size of the scanning probe, a maximum of 10% of the population at risk was used [20]. The maps were constructed using the QGIS, version 3.4.4 software, which is available to researchers. The analytical approach adopted was adequate to fulfill the study objectives and has proved to be robust in other studies [21–24].

The research fulfilled the determinations described in Resolution 466/2012 of the National Health Council and followed the Declaration of Helsinki. The study was approved by the Health Ministry of Misiones, Argentina, as well as by the Ethics Committee for Research involving Human Subjects in Brazil, under CAAE number 84371518.0.0000.0107.

## Results

In the state of Paraná, 71.2% of the municipalities had a hyperendemic incidence rate, that is, above 40 cases per 100,000 inhabitants. In the state of Santa Catarina, the hyperendemic situation affected 20.7% of the municipalities, and in Rio Grande do Sul 9.5%. In Argentina, the Misiones province presented a situation of hyperendemicity in 41.2% of the Departments (Table 1).

**Table 1:** Distribution of municipalities/departments classified according to incidence rate, in the states of Paraná, Santa Catarina and Rio Grande do Sul, Brazil and in Misiones province, Argentina in the period 2010 to 2016.

	Paraná		Santa Catarina		Rio Grande do Sul		Misiones	
	*n*	%	*n*	%	*n*	%	*n*	%
Hyperendemic	284	71.2	61	20.7	47	9.5	7	41.2
Very high	39	9.8	40	13.6	30	6	7	41.2
High	18	4.5	45	15.2	63	12.7	2	11.8
Medium	16	4.0	39	13.2	70	14	0	0
Low	0	0	3	1	2	0.4	1	5.9
No cases	42	10.5	107	36.3	285	57.3	0	0

Note: Classification of incidence rates—low (less than 2.00), medium (2.00 to 9.99), high (10.00 to 19.99), very high (20.00 to 39.99) and hyperendemic situation (greater than or equal to 40.00), Ministry of Health of Brazil, (2017).

**Fig 2:**
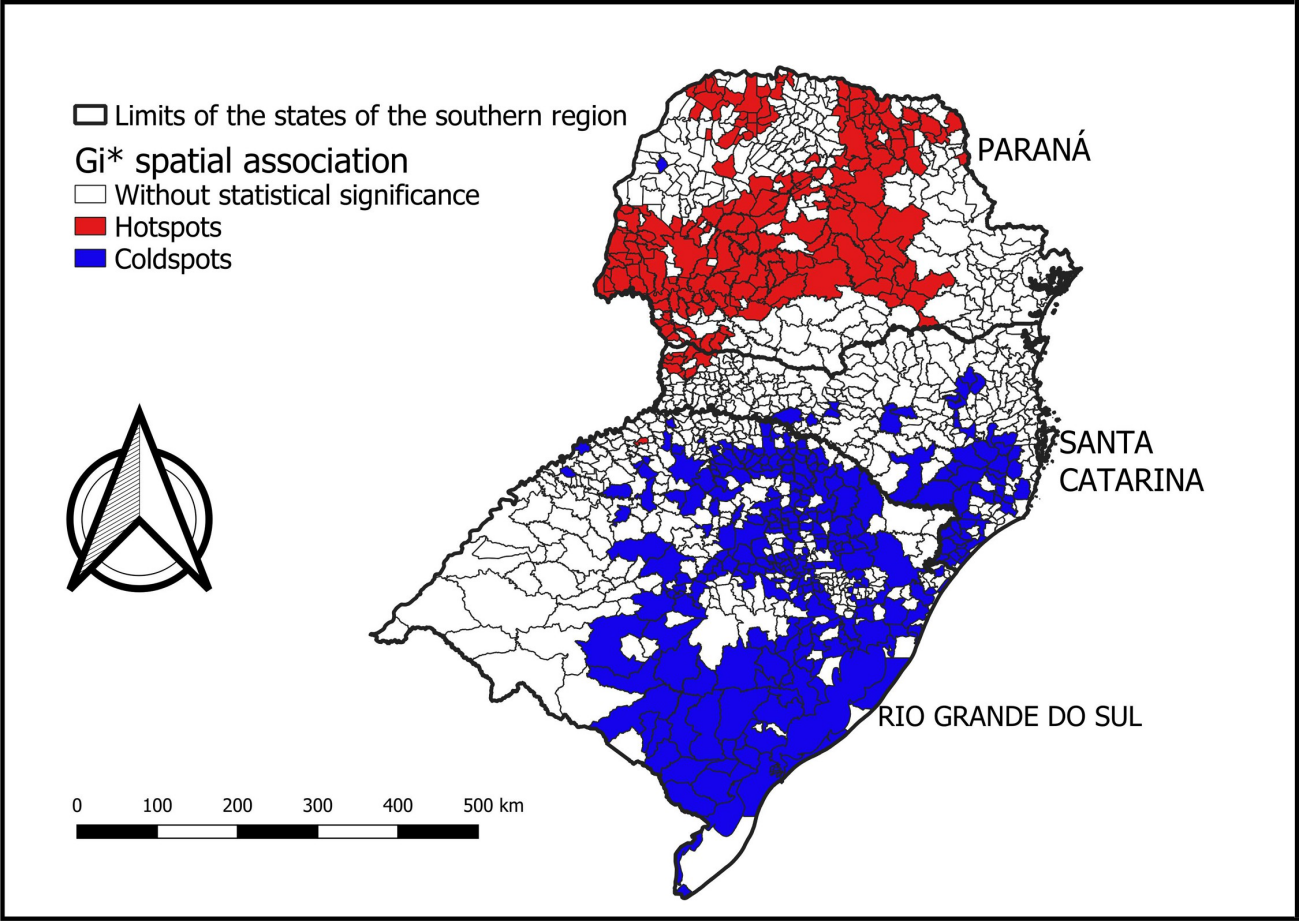
Areas of spatial association according to the Gi* technique, showing hotspots and coldspots for the incidence of leprosy in southern Brazil, in the period 2010 to 2016.

As shown in Fig 2, the GI* statistic identified clusters of high incidence rates in a large area of the state of Paraná and in some municipalities in the western region of Santa Catarina, as well as low-risk clusters in much of the state of Rio Grande do Sul, which demonstrates that the proportion of distributions of rates of new leprosy cases, shown in Table 1, presents a spatial association as shown in Fig 2.

**Fig 3:**
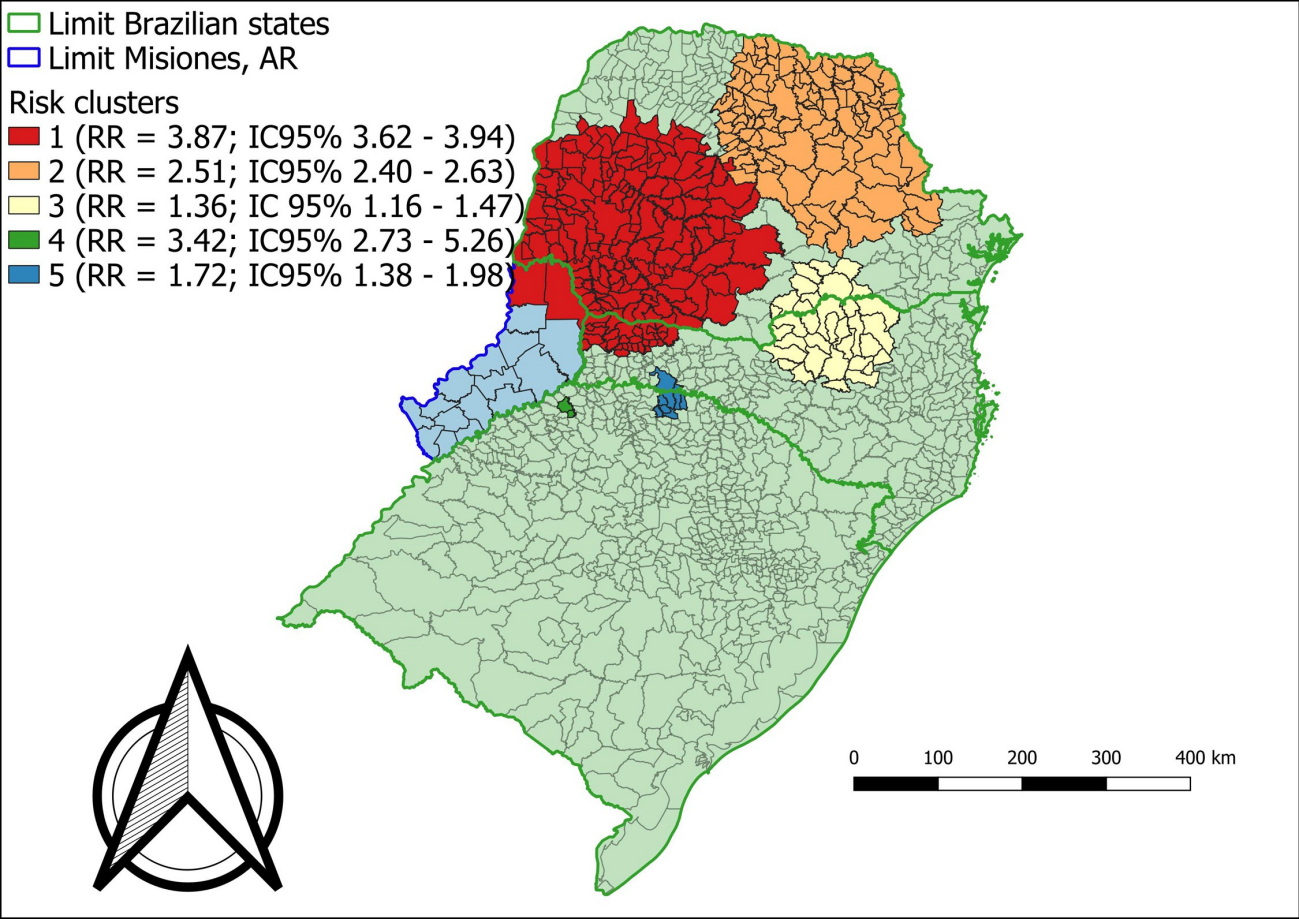
Areas of relative risk for new cases of leprosy in Southern Brazil and Misiones—Argentina, 2010 to 2016.

When starting the scan statistic, it was found that, in the analysis of the entire scenario, the concentration of high municipal rates in the state of Paraná “hid” municipalities with high rates in other territories. This is due to the RR calculation taking into account the total population and the affected population, which allows for a certain dilution of the RR in some locations. The differentiated analysis allowed the verification that the state of Paraná statistically interfered in the RR of the other locations studied (Figs 2 and 3).

**Fig 4:**
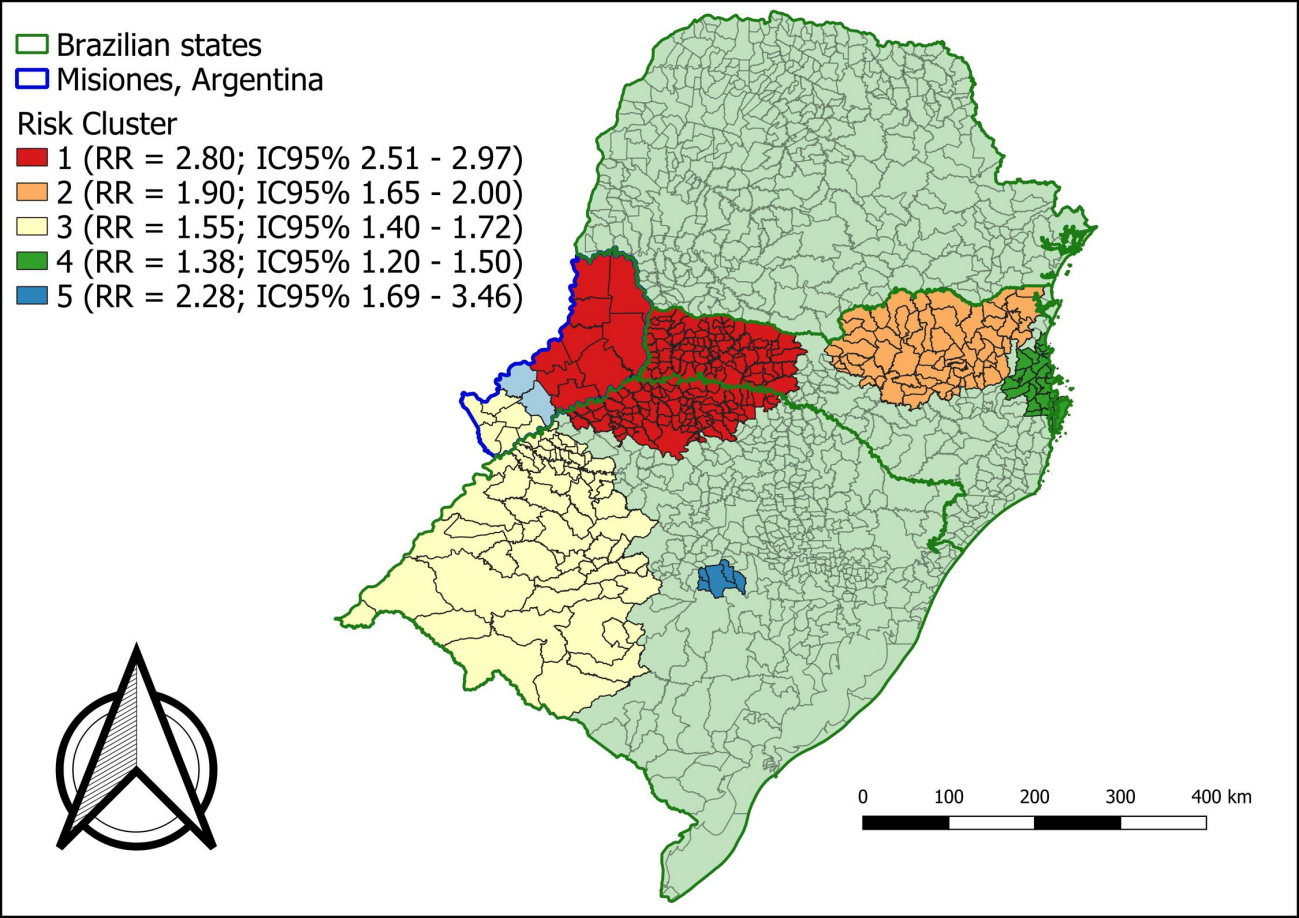
Areas of relative risk for new cases of leprosy in Southern Brazil (excluding Paraná) and Misiones, Argentina, 2010 to 2016.

To better understand this influence, Table 2 presents the general description of each analysis. Through the information in this table, it is possible to identify how Paraná exerts an influence over the entire analyzed region. While the state has a population that represents just over a third of the total studied, it accounts for approximately two thirds of the cases during the period. This characteristic of Paraná demonstrates the reason why the spatial analysis showed a difference according to its presence or absence as a variable. Furthermore, with the state of Paraná, the annual incidence rate is 5.2 new cases per 100,000 inhabitants, whereas, without including it in the analysis, the rate is 2.4 per 100,000. Tables 3 and 4 present the details of each risk area identified in Figs 3 and 4.

**Table 2:** General description of the relative risk analyses for new cases of leprosy in the southern region of Brazil and in the province of Misiones, Argentina, 2010 to 2016.

Areas analyzed	PR/SC/RS/MI	SC/RS/MI
Period	2010 to 2016	2010 to 2016
Number of municipalities / departments	1.207	808
Total resident population	28,519,550	18,100,577
Total cases for the period	10,320	3.074
Annual incidence rate	5.2 / 100,000	2.4 / 100,000
Number of risk clusters	5	5

Legend: PR–Paraná, SC–Santa Catarina, RS–Rio Grande do Sul, MI–Misiones.

**Table 3:** Description of areas of relative risk for new cases of leprosy in Southern Brazil and Misiones, Argentina, 2010 to 2016.

Paraná / Santa Catarina / Rio Grande do Sul / Misiones									
Cluster	Step*	Radius (Km)	Population	Cases	AR	RR	CI 95%	Highest RR	p value
1**	4	168.76	2,879,128	3.073	15.6	3.87	3.62 to 3.94	5	< 0.0001
2	7	177.33	2,810,478	2.225	11.3	2.51	2.40 to 2.63	3	< 0.0001
3	5	85.52	601	283	7	1.36	1.16 to 1.47	2	< 0.0001
4	2	14.03	26	36	17.6	3.42	2.73 to 5.26	3	0.0001
5	2	26.08	203	121	8.9	1.72	1.38 to 1.98	2	0.0024

Label: AR—Annual incidence rate for the Cluster; RR—Relative Risk; CI–Confidence Interval.

* Number of layers/relative risk levels within each cluster.

** If the cluster has departments in Argentina.

**Table 4:** Description of areas of relative risk for new cases of leprosy in the states of Santa Catarina, Rio Grande do Sul and Misiones, Argentina, 2010 to 2016.

Santa Catarina/ Rio Grande do Sul/ Misiones									
Cluster	Step*	Radius (km)	Population	Cases	AR	RR	CI 95%	Highest RR	p value
1**	11	179.92	1,847,575	728	5.8	2.8	2.51 to 2.97	10	<0.0001
2	6	139.05	1,752,448	502	4.3	1.9	1.65 to 2.00	9	<0.0001
3**	7	295.04	1,749,996	438	3.6	1.55	1.40 to 1.72	4	<0.0001
4	2	60.37	1,555,194	345	3.2	1.38	1.20 to 1.50	1	0.00049
5	3	26.53	73.54	30	5.5	2.28	1.69 to 3.46	4	0.0170

Label: AR—Annual incidence rate for the Cluster; RR—Relative Risk; CI–Confidence Interval.

* Number of layers/relative risk levels within each cluster.

** Cluster with departments in Argentina.

Figs 3 and 4 show risk areas identified by the scan statistic, covering municipalities in southern Brazil and in the province of Misiones, Argentina. It was possible to verify a different pattern when the analysis of all the municipalities together was compared to the analysis without the state of Paraná. The municipalities of Santa Catarina and Rio Grande do Sul present more risk areas when analyzed together with the province of Misiones, Argentina; however, excluding the state of Paraná.

In the first assessment identified in Fig 3, five risk areas were highlighted with an RR above 1 and a significant *p* value. These areas were biased towards the Paraná region. In the second analysis, represented in Fig 4, excluding the state of Paraná, there are other areas, extending to the coast of the state of Santa Catarina and the interior of the state of Rio Grande do Sul. In both analyses, Misiones’ departments were found in some risk areas. It is also important to highlight that in the first analysis, Fig 3 (including the state of Paraná), only one risk area was identified including at least two departments of Misiones–Argentina, these being the General Manuel Belgrano and Iguazu departments bordering Brazil.

The second analysis in Fig 4 made it possible to identify two risk areas (clusters), involving almost all the 17 departments of Misiones, Argentina (except for the San Ignácio and Oberá departments). Cluster 1, when analyzed excluding the state of Paraná, comprised an extensive territorial space, involving the limits of the province of Misiones, Argentina, which borders the territories of Paraguay to the west and the states of the southern region of Brazil to the east.

In Table 3, a risk cluster with an RR of 3.87 and a significant *p* value was observed, and in Table 4 (excluding Paraná) a risk cluster with an RR of 2.80 (*p* < .0001) was observed. Furthermore, in Tables 3 and 4, higher RR values within the clusters are indicated (which is a possibility of the isotonic technical approach based on discrete Poisson distribution), which means that the RR would not be uniform across the cluster, however, would have a higher RR center. The results show that, in some cases, the highest RR represents more than twice the RR of the total cluster (example: Table 3, clusters 1 and 2), usually when the cluster is large, which would allow for several layers. In other cases, the highest RR is very close to the total RR, which occurs in smaller clusters (example: Table 3, cluster 4 and 5).

## Discussion

The detection of new cases of leprosy in Latin America reached 2.7 cases per 100,000 inhabitants, making it the second highest region in the world with the highest number of leprosy cases. In this geographic space, Brazil is accounts for approximately 92% of cases, followed by Paraguay and Argentina. Due to the large proportion of leprosy cases, Brazil was included in the list of 22 priority nations around the world for the control of the disease [2,25].

In Argentina, leprosy was eliminated as a public health problem in 2006. However, Brazil, despite the substantial reduction in the number of cases (37.7%) between 2009 and 2020, has not yet reached the elimination stage as recommended by the WHO [26,27]. The disease presents heterogeneity in its territorial distribution, a phenomenon recognized by the WHO and observed in the Southern Region of Brazil [3,28], which has low case rates. However, this study shows that the frequent occurrence of cases is still persistent in many municipalities, with high incidence rates observed mainly in the state of Paraná, Brazil and also in the province of Misiones, Argentina, where hotspots, classified with very high and hyperendemic incidence rates, were found.

In 1995, Rio Grande do Sul became the first state in Brazil to achieve the goal of eliminating leprosy as a public health program. It currently has a situation of low endemicity and in 2019 less than 100 cases of the disease were reported [4,29]. In this study, Rio Grande do Sul, Brazil had a smaller number of municipalities with a high incidence rate of leprosy, however, when assessed individually, it still shows regions for concern, with 140 municipalities presenting very high and hyperendemic rates. These also appear in several clusters with a relative risk for new cases of leprosy (Fig 2).

In situations of a reduction in new cases, the monitoring of the contacts of people affected by leprosy, which constitutes the group that has a higher risk of illness when compared to the general population, becomes essential for disease surveillance actions [4]. An analysis in 2020 indicated relevant information regarding the state of Rio Grande do Sul, Brazil, with this being the only state among the states of the southern region of Brazil with a worsening in the indicator of the proportion of contacts examined among new cases, in the year 2019 compared to the year 2012 [4]. Furthermore, it is possible to observe in the maps presented in Figs 2–4, two distinct scenarios, as the hyperendemic situation in most of Paraná, Brazil was masking other risk areas in other locations. When the RR map was analyzed without the state of Paraná - Brazil, it was possible to identify more risk clusters in Rio Grande do Sul, Brazil, Santa Catarina, Brazil and in the province of Misiones, Argentina, which did not appear before due to the endemic influence of leprosy in Paraná, Brazil, showing the spatial heterogeneity in the occurrence of the disease [30].

These findings indicate the relevance of studies on the spatial distribution of leprosy that show the geographic portrait of the endemic, which would not be perceived with tabular data alone [31]. It is therefore possible to infer the influence of a hyperendemic territory on surrounding territorial spaces. Knowledge about the risk of contracting leprosy in the presented clusters shows that the disease is “spatially determined” [32] and involves migratory and socioeconomic processes, demonstrating that the endemic is not sustained only by microspatial determinants. It can also be considered that these cluster regions are mostly composed of small municipalities that have different relationships between them, which facilitate the movement of people. This work highlights that some parts of these risk clusters occur on borders between countries. Accordingly, the risk areas for new cases of leprosy show the existing interdependence between states/provinces and neighboring countries. This condition is characteristic of border regions, with intense daily movement of people due to work, education, consumption, and access to public services, particularly public health services [9,15]. Therefore, it is essential to study and share information between bordering countries to enable articulation and joint planning aimed at controlling diseases, especially infectious contagious ones, such as leprosy [10].

Considering these integration experiences, it is important to highlight the clusters found in Misiones, Argentina, especially when evaluated without the state of Paraná, Brazil. Through this analysis, it was possible to visualize an interaction with many departments in the province of Misiones in Argentina with RRs for new cases of leprosy. This evidence is in agreement with the findings of Odriozola et al., (2017) [5] for the province of Corrientes and [33] for the province of Formosa, both in Argentina.

Although Argentina is considered a country that has achieved the goal of elimination, the distribution of the disease is not uniform, with it still being a public health problem in the northern region of the country [5]. The results of this study indicate that the province of Misiones and the provinces of Corrientes and Formosa [5,33], located in northern Argentina bordering the southern region of Brazil and/or Paraguay, are also regions with a relevant increase in the annual incidence of cases in Argentina. This epidemiological condition may be configuring itself as endemic areas, possibly due to the influence of two main factors; the reduction in the active search for cases by health services, since the disease is not considered a health problem in the country, despite being endemic in these provinces, and because these provinces (Misiones and Corrientes) border Brazil, where there is a high rate of detection of new cases [3,5,33].

To comprehend the magnitude of the disease in a given territory, it is necessary to study spatially represented historical series, relating to the accumulation of cases (prevalence) in a given geographic space, to confirm whether the transmission strength (detection coefficient) is decreasing due to the reduction in cases or because surveillance, control and diagnostic measures have been relaxed. According to the Global Leprosy Strategy 2016–2020, the case detection coefficient is an important monitoring indicator [34], as the emergence of new cases will continue to occur at a relevant frequency, even in those places that were endemic and that managed to achieve leprosy-free status. Therefore, the analysis of the detection coefficient in a region or clusters is an important strategy for recognizing the endemic profile of the disease [35].

The existence of geographic and political-administrative borders between countries are not perceived by the populations residing in these areas, due to the cultural and economic interdependence among them, turning these territories into unique urban conglomerates, composed by characteristics of both countries. In these places, leprosy has a profile due to the circulation of *Mycobacterium leprae*, especially due to socioeconomic vulnerability characteristic attributes of the person affected by leprosy and the territory [9].

The study of leprosy in these South American territories, through the use of spatial analysis techniques, helped to identify risk areas and, therefore, priority areas within the studied regions, highlighting the important applications for public health and for the identification of spatial patterns of leprosy. This study advances the knowledge on the distribution of leprosy in the southern region of Brazil and in the Misiones province of Argentina, geographically close regions that need to implement health policies to control the disease. These should be directed toward constant monitoring of a cluster of RR for new cases, considering the socioeconomic differences of the states/provinces/municipalities/departments of each country and interregional differences, through epidemiological surveillance and care for contacts of people who have been diagnosed with leprosy in the region.

The study brought important findings for comparison with the global health goals for leprosy agreed by the WHO, especially those related to the reduction of new cases and the number of countries with autochthonous cases. A decrease in autochthonous cases was confirmed in Misiones in Argentina and in the southern region of Brazil, however, the existence of risk clusters for the occurrence of new cases involving both countries cause concern.

As a limitation of the study, it is important to consider that with the analysis techniques used it was not possible to state that the endemic behavior of the disease in Misiones, Argentina is influenced only by the high prevalence of cases in Brazil. Furthermore, the data used came from official databases of the Health Ministries of Brazil and Argentina, with these databases being provided by municipal and departmental health units, subject to the presence of incompleteness in data. Additionally, it is necessary to emphasize that the existence of undiagnosed cases is possible, due to the long asymptomatic and/or oligosymptomatic stage of the disease, which is usually more likely to be noticed by specialists trained to detect these events [36].

## Conclusions

There were hyperendemic incidences in all regions of the study, with higher rates in the state of Paraná. Areas of RR above 1 and significant *p* value were found in most of Paraná, on the coast of Santa Catarina, in the interior of Rio Grande do Sul and in the province of Misiones in Argentina. The findings indicate a high probability of similar illness in adjacent areas, according to their relative position in space, as the occurrence of leprosy in a determined region can be influenced by the infection rates of neighboring clusters. With so many differences and particularities still little known and studied, it is necessary to implement and improve public policy in border regions in order to meet the challenges of border flows, health security and integration between neighboring countries.
